# Dimethylthiourea Alleviates Drought Stress by Suppressing Hydrogen Peroxide-Dependent Abscisic Acid-Mediated Oxidative Responses in an Antagonistic Interaction with Salicylic Acid in *Brassica napus* Leaves

**DOI:** 10.3390/antiox11112283

**Published:** 2022-11-18

**Authors:** Bok-Rye Lee, Van Hien La, Sang-Hyun Park, Md Al Mamun, Dong-Won Bae, Tae-Hwan Kim

**Affiliations:** 1 Grassland Science Laboratory, Department of Animal Science, Institute of Agricultural Science and Technology, College of Agriculture & Life Science, Chonnam National University, Gwangju 61186, Republic of Korea; 2 Institute of Environmentally-Friendly Agriculture (IEFA), Chonnam National University, Gwangju 61186, Republic of Korea; 3 Center of Crop Research for Adaption to Climate Change (CRCC), Thai Nguyen University of Agriculture and Forestry, Thai Nguyen 24000, Vietnam; 4 Central Instrument Facility, Gyeongsang National University, Jinju 52828, Republic of Korea

**Keywords:** abscisic acid, dimethylthiourea, drought stress, redox signaling, salicylic acid

## Abstract

In plants, prolonged drought induces oxidative stress, leading to a loss of reducing potential in redox components. Abscisic acid (ABA) is a representative hormonal signal regulating stress responses. This study aimed to investigate the physiological significance of dimethylthiourea (DMTU, an H_2_O_2_ scavenger) in the hormonal regulation of the antioxidant system and redox control in rapeseed (*Brassica napus* L.) leaves under drought stress. Drought treatment for 10 days provoked oxidative stress, as evidenced by the increase in O_2_^•−^ and H_2_O_2_ concentrations, and lipid peroxidation levels, and a decrease in leaf water potential. Drought-induced oxidative responses were significantly alleviated by DMTU treatment. The accumulation of O_2_^•−^ and H_2_O_2_ in drought-treated plants coincided with the enhanced expression of the *NADPH oxidase* and *Cu/Zn-SOD* genes, leading to an up-regulation in oxidative signal-inducible 1 (*OXI1*) and mitogen-activated protein kinase 6 (*MAPK6*), with a concomitant increase in ABA levels and the up-regulation of ABA-related genes. DMTU treatment under drought largely suppressed the drought-responsive up-regulation of these genes by depressing ABA responses through an antagonistic interaction with salicylic acid (SA). DMTU treatment also alleviated the drought-induced loss of reducing potential in GSH- and NADPH-based redox by the enhanced expression of glutathione reductase 1 (*GR1*) and up-regulation of oxidoreductase genes (*TRXh5* and *GRXC9*). These results indicate that DMTU effectively alleviates drought-induced oxidative responses by suppressing ABA-mediated oxidative burst signaling in an antagonistic regulation of SA.

## 1. Introduction

Plants exposed to various stressors commonly generate reactive oxygen species (ROS) as one of the earliest responses of plant cells. Drought, the most common environmental stress, provokes water loss in photosynthetic tissues [1,2], leading to stomatal closure [3]. Drought-responsive stomatal closure decreases the internal CO_2_/O_2_ ratio, which is responsible for ROS generation via photorespiration [4,5].

Excessive generation of ROS damages proteins, lipids, RNA, and DNA [5,6,7]. ROS are also involved in regulating stress responses in plants as signal transduction molecules [2,8,9]. Therefore, the level of ROS in plant cells requires tight regulation by the enzymes involved in ROS scavenging and generation, such as peroxidases (POXs), NADPH oxidase, superoxide dismutase (SOD), and catalase (CAT) [10,11,12], as well as by non-enzymatic metabolic pathways (e.g., glutathione-ascorbate cycle) [13,14]. Moreover, excessive accumulation of ROS disrupts the cellular redox balance of GSH-based (GSH/GSSG) and NAD(P)H-based [NAD(P)H/NAD(P)^+^] [5,6,11,13]. The oxidative signal-inducible 1 gene (OXI1), which encodes a serine/threonine kinase, is induced by a wide range of H_2_O_2_-generating stimuli [15]. OXI1 activates a mitogen-activated protein kinase (MAPK) cascade (MAPK3/6) and induces different transcription factors that regulate the ROS-scavenging and ROS-producing pathways [16].

Since plant stress stimuli modify the endogenous level of ROS and hormones and generally up-regulate oxidative burst and hormonal signaling [2,17,18], the interactive regulation between ROS and hormones in plant stress responses and tolerance has been widely studied [14,18,19,20]. Among these interactions, an interplay between hydrogen peroxide (H_2_O_2_) and ABA has been widely studied under different environmental stresses. For instance, the ABA signal increases respiratory burst oxidase homolog (NADPH oxidase) expression, leading to H_2_O_2_ production, which activates ABA signaling [9]. This feed-forward loop between ABA and H_2_O_2_ activates the plasma membrane Ca^2+^ channels [2,21,22]. In addition, ABA-mediated H_2_O_2_ accumulation up-regulates the genes encoding antioxidative enzymes, such as superoxide dismutase (SOD), catalase (CAT), and peroxidase (POX) [23,24,25,26]. Thus, ROS (particularly H_2_O_2_) are considered a part of ABA signaling [9,27,28]. Furthermore, ROS-mediated SA biosynthesis via the Ca^2+^ signal [29,30] and SA-induced ROS accumulation are involved in plant stress responses and tolerance. Biotic and abiotic stressors stimulate a positive feed-forward loop between H_2_O_2_ and SA through the mutual activation of their biosynthesis [30]. The time-course analysis in *Arabidopsis* has shown that the crosstalk between H_2_O_2_ and SA has a much earlier peak time than that of ABA [20]. Our previous studies have shown that SA-stimulated H_2_O_2_ accumulation and SA responses during the early drought phase are a part of upstream H_2_O_2_-stimulated ABA accumulation, which causes ABA signaling and responses, leading to severe drought symptoms during the late phase [2]. Various studies have assessed the alleviating effects of ROS scavenger, such as imidazole, tiron, and dimethylthiourea (DMTU), on oxidative responses induced by various stresses [31,32,33]. However, many questions remain unresolved regarding the interaction between hormones and H_2_O_2_ (including possible antagonistic responses to DMTU) in ROS management and redox control in terms of the stress tolerance process.

In this study, we addressed the physiological significance of DMTU (an H_2_O_2_ scavenger) for drought stress responses in rapeseed (*Brassica napus* L.) leaves. We focused on DMTU-induced drought resistance in the hormonal regulation of the antioxidant system, ROS signaling, redox control, and their interactions. Therefore, this research was designed with three groups: control, drought, and drought with DMTU treatment for 10 days.

## 2. Materials and Methods

### 2.1. Plant Growth and Treatment

*Brassica napus* L. (cv. Capitol) seeds were sown in bed soil in a tray. When the plants were at the four-leaf stage, the seedlings were transferred to 2-L pots containing a mixture of soil and perlite (70:30, *w*/*w*) in a greenhouse. A complete nutrient solution was continuously supplied to the plants [1]. Metal halide lamps (400 µmol photons m^−2^ s^−1^ at the canopy height for 6 h per day) were used to supply natural light. Plants were selected based on morphological similarity after six weeks and divided into three groups. The first group was irrigated with 200 mL water for the well-watered plants (control), the second group received 20 mL water (drought), and the third group was foliar-sprayed with 500 µM DMTU daily under the drought-stressed condition (drought + DMTU) for 10 days. Sampling was performed at 0, 5, and 10 days after treatment. In this study, the mature leaves ranked 4–12 (i.e., rank one for the oldest leaf) were considered. After sampling, leaf tissues were cut and frozen immediately in liquid nitrogen and stored in a deep freezer (−80 °C) until further analysis. The part of data in control and drought-alone-treated plants was previously published by Lee et al. [18].

### 2.2. Measurement of Leaf Water Potential (Ψ_w_)

The leaf water potential (*Ψ_w_*) was evaluated according to the petiole xylem-pressure potential and measured using a pressure chamber (PMS Instruments, Corvallis, OR, USA). The leaf water status measurements were taken before dawn on the first or second fully expanded green leaf proximal to the petiole apex.

### 2.3. ROS and MDA Concentrations

Fresh samples (0.5 g) were mixed with 1.5 mL of 50 mM KPO_4_^−^ buffer (pH 7.0) and centrifuged at 10,000× *g* for 25 min at 4 °C. After centrifugation, the supernatants were used to determine the superoxide anion radical (O_2_^•−^) and H_2_O_2_ concentration. The O_2_^•−^ concentration was measured by hydroxylamine oxidation [7]. The H_2_O_2_ concentration was measured colorimetrically as described by Lee et al. [34] using titanium sulfate. The H_2_O_2_ concentration was calculated using the extinction coefficient 0.28 mM^−1^ cm^−1^ and expressed as nmol g^−1^ tissue fresh weight. The lipid peroxidation level was determined by measuring the concentration of malondialdehyde (MDA) as described previously [7].

### 2.4. Redox Status Analysis

To extract glutathione, approximately 200 mg of fresh leaves were homogenized in 5% of 5-sulfosalicylic acid and centrifuged at 12,000× *g* for 10 min at 4 °C. Glutathione content was determined by microplate assay using the GSH/GSSG Kit GT40 (Oxford Biomedical Research Inc., Rochester Hills, MI, USA). The determination of oxidized and reduced pyridine nucleotide content was conducted as described by La et al. [17]. For the NADP^+^ and NADPH extraction, 200 mg fresh leaves were homogenized with 0.8 mL of 0.2 N HCl and 0.2 M NaOH, respectively. One hundred microliters of the extracts were heated at 95 °C for 1 min, and the reactions were stopped in an ice bath. For the NADP^+^ assay, the supernatant was neutralized by 0.2 M NaOH to a final pH of 5–6, and the NADPH solution was neutralized by 0.2 N HCl to a final pH of 7–8. Forty microliters were added to the reaction mixture containing 0.1 M HEPES (pH 7.5) that consisted of 2 mM Na_2_EDTA, 1.2 mM dichlorophenolindophenol, 20 mM phenazine methosulfate, and 10 mM glucose-6-phosphate. The reaction was started by adding 2 μL glucose 6-phosphate dehydrogenase. The concentrations of NADP^+^ and NADPH were determined from the standard curve using 1–100 pmol contents.

### 2.5. Phytohormone Analysis

Quantitative analysis of phytohormones in the leaf tissue was performed according to the methodology described by La et al. [17]. Fifty milligrams of fresh leaves in a 2 mL tube were frozen in liquid nitrogen and ground using a Tissuelyser II (Qiagen, Hilden, Germany). The ground sample was subjected to extraction with 500 µL of the extraction solvent (2-propanol/H_2_O/concentrated HCl [2:1:0.002, *v*/*v*/*v*)]) containing d6-ABA and d6-SA as the internal standard (50 ng) for ABA and SA, respectively, for 24 h at 4 °C. Dichloromethane (1 mL) was added to the supernatant, and this mixture was then centrifuged at 13,000× *g* for 5 min at 4 °C. The lower phase, poured into a clean screw-cap glass vial, was dried under nitrogen and dissolved in pure methanol. The completely dissolved extract, ensured by vortexing and sonicating, was transferred to a high-performance liquid chromatography (HPLC) vial. Hormones were analyzed using the HPLC electrospray ionization tandem mass spectrometry (HPLC-ESI-MS/MS) method by a reverse phase C18 HPLC column. The chromatographic separation of hormones and their internal standard from the plant extracts was performed on an Agilent 1100 HPLC (Agilent Technologies, Santa Clara, CA, USA), Waters C18 column (150 × 2.1 mm, 5 µm), and API3000 MS-MRM (Applied Biosystems, Waltham, MA, USA).

### 2.6. Isolation of Total RNA and RT-qPCR Analysis

Total RNA was isolated from 100 mg leaf tissue using the RNAiso Plus reagent (Takara, Nojihigashi 7-4-38 Kusatsu, Shiga, Japan). The GoScript Reverse Transcription System (Takara) was used to synthesize cDNA from RNA. RT-qPCR reactions were carried out on a BioRad CFX96 qPCR System using the TB Green Premix Ex Taq (Takara). PCR reactions were initiated at 95 °C for 5 min; afterward, 45 cycles were initiated at 95 °C for 30 s, 51–60 °C for 30 s (depending on the target primers), 72 °C for 30 s, and a final extension at 72 °C for 5 min. The RT-qPCR reactions were performed in duplicate for each of the three independent samples. Primer sequences used for RT-qPCR are presented in Supplementary Table S1. All quantifications were normalized to actin.

### 2.7. Statistical Analysis

A completely randomized design was used with three replicates per treatment. Duncan’s multiple range test was used to compare the means of separate replicates. Statistical significance was postulated at *p* < 0.05. Statistical analysis of all measurements was performed using SAS 9.1.3 software (SAS Institute Inc., Cary, NC, USA). Heatmap, correlation coefficient analysis, and principal component analysis (PCA) were conducted using MetaboAnalyst 4.0 (http://www.metaboanalyst.ca, accessed on 1 May 2022).

## 3. Results

### 3.1. Leaf Water Potential and Lipid Peroxidation Level

**Figure 1:**
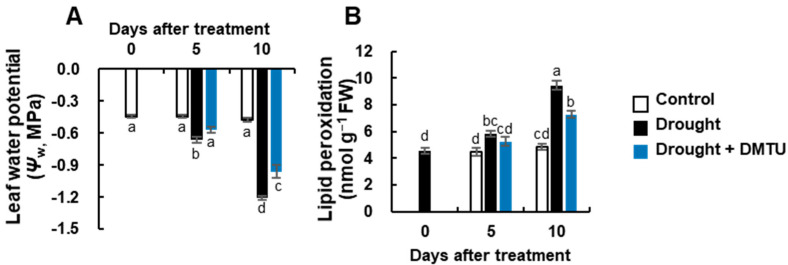
The changes in leaf water potential (*Ψ_w_*, (**A**)), and lipid peroxidation (**B**) level in the leaves of *Brassica napus* under control, drought, or drought with dimethylthiourea (Drought + DMTU) treatments for 10 days. Results are represented as mean ± SE for *n* = 3. Means with different letters are significantly different at *p <* 0.05, according to Duncan’s multiple range test.

The changes in leaf water potential (*Ψ_w_*) and lipid peroxidation level as affected by drought alone (drought) or DMTU application under drought (drought + DMTU) are presented in Figure 1. The *Ψ_w_* significantly decreased to −1.20 MPa in the drought alone condition but was less affected by DMTU treatment under the drought condition (−0.9 MPa) for 10 days (Figure 1A). The concentration of MDA, as a marker of lipid peroxidation caused by oxidative stress, also significantly increased by 1.9- and 1.5-fold, respectively, in the drought and DMTU under drought treatments for 10 days, as compared to that of the control plant (Figure 1B).

### 3.2. ROS Status and ROS Signaling Genes Expression

**Figure 2:**
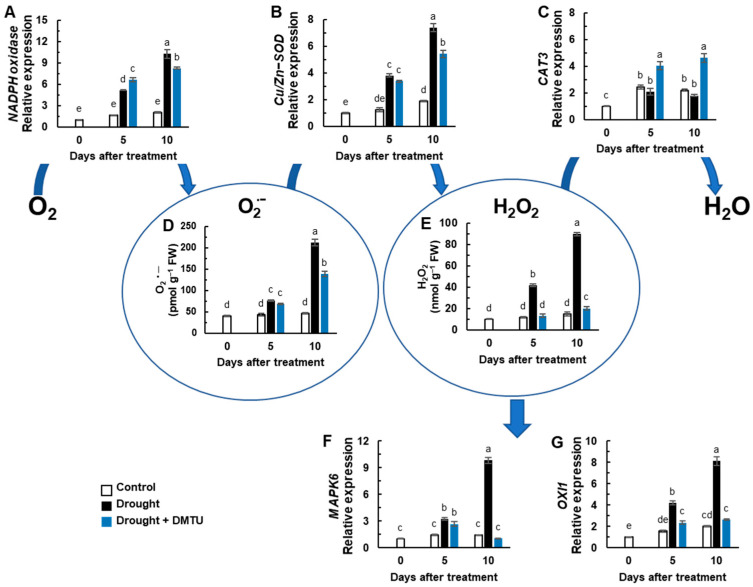
The changes in the concentration of reactive oxygen species (ROS) and the expression of ROS synthesis and signaling-related genes in the leaves of *Brassica napus* under control, drought, or drought with dimethylthiourea (Drought + DMTU) treatments for 10 days. Expression of (**A**) *NADPH oxidase*, (**B**) superoxide dismutase (SOD)-related gene, *Cu/Zn-SOD*, (**C**) catalase 3 (CAT3), (**F**) transcription factor, *MAPK6*, and (**G**) *oxidative signal-inducible* (*OXI1*), and the concentration of (**D**) O_2_^•−^ and (**E**) H_2_O_2_. The transcriptional levels in control plants at day 0 were set to 1. Results are represented as mean ± SE for *n* = 3. Means with different letters are significantly different at *p <* 0.05, according to Duncan’s multiple range test.

The expression of *NADPH oxidase (RbohD)* and Cu/Zn superoxide dismutase encoding gene (*Cu/Zn-SOD*) was significantly enhanced in drought with or without DMTU treatment (Figure 2A,B). The expression of the catalase-encoding gene *CAT3* was significantly enhanced only in drought with DMTU treatment, showing a 4.6-fold increase (Figure 2C). The O_2_^•−^ concentration gradually increased under drought stress but was reduced by DMTU treatment (Figure 2D). The H_2_O_2_ concentration in the drought-alone treatment significantly increased by 3.6- and 6.0-fold on days 5 and 10, respectively, compared to the control. However, there was a 1.3-fold increase in DMTU-treated plants only at day 10 (Figure 2E). Expressions of ROS-responsive signaling-related protein kinase, mitogen-activated protein kinase 6 (*MAPK6*), and *OXI1* were significantly up-regulated in the drought-alone treatment but were suppressed by DMTU treatment (Figure 2F,G).

### 3.3. Endogenous ABA and SA Status, ABA- and SA-Synthesis, and Signaling Genes Expression

**Figure 3:**
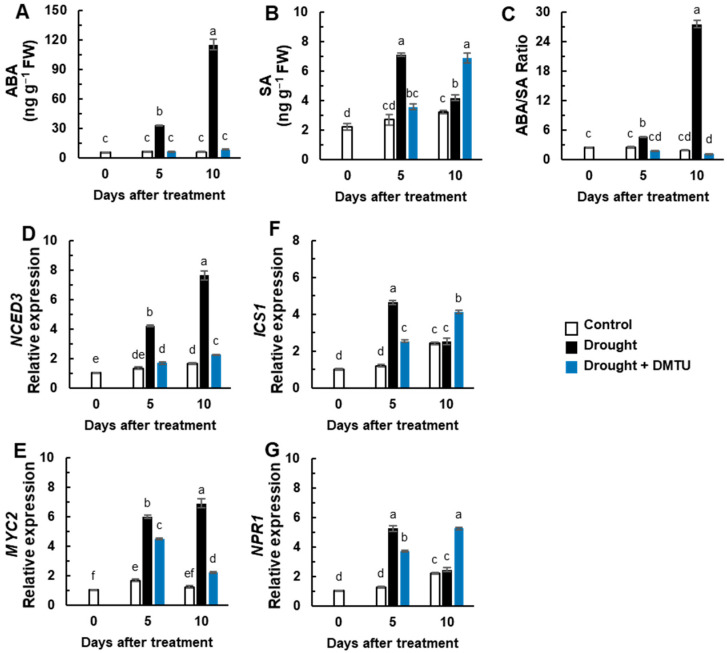
The changes in the levels of endogenous hormones and expression of hormone synthesis or signaling-related genes in the leaves of *Brassica napus* under control, drought, or drought with dimethylthiourea (Drought + DMTU) treatments for 10 days. (**A**) abscisic acid (ABA) and (**B**) salicylic acid (SA) concentrations, and (**C**) the ratio of ABA to SA. The expression of the (**D**) ABA synthesis-related gene *NCED3*, (**E**) ABA signaling-related gene *MYC2*, (**F**) SA synthesis-related gene *ICS1*, and (**G**) SA signaling-related gene *NPR1*. The transcriptional levels in control plants at day 0 were set to 1. Results are represented as mean ± SE for *n* = 3. Means with different letters are significantly different at *p <* 0.05, according to Duncan’s multiple range test.

Endogenous ABA levels gradually increased up to 19.4-fold in the drought alone treatment compared to that of the control plants, whereas it was not changed in the DMTU treatment in the drought condition (Figure 3A). SA levels significantly increased only at day 5 in the drought-alone treatment and day 10 in the DMTU treatment in the drought condition (Figure 3B). The resulting ABA/SA ratio was remarkably enhanced by the drought-alone treatment but greatly depressed by the DMTU treatment in the drought condition (Figure 3C).

The expressions of a gene associated with ABA synthesis, 9-cis-epoxycarotenoid dioxygenase (*NCED3*), and an ABA signaling-related gene, transcriptional factor *MYC2*, were enhanced more than 4.2-fold in the drought alone condition, whereas their expression levels were alleviated by DMTU treatment (Figure 3D,E). The expression of the SA synthesis gene, iso-chorismate synthase 1 (*ICS1*), and a SA regulatory gene, non-expression of the pathogenesis-related gene (*NPR1*), were up-regulated in the drought condition with or without DMTU at day 5, but this expression was only maintained at a high level in the presence of DMTU under the drought condition (Figure 3G).

### 3.4. Redox Changes and the Expression of Redox Signaling Genes

**Figure 4:**
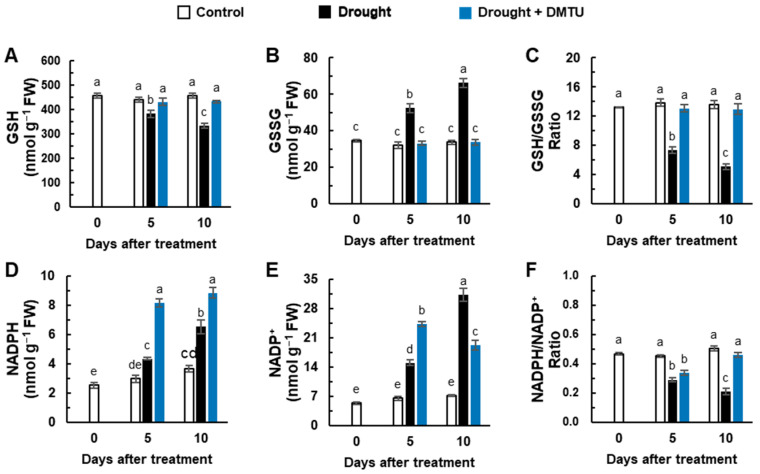
The changes of redox status in the leaves of *Brassica napus* under control, drought, or drought with dimethylthiourea (Drought + DMTU) treatments for 10 days. The concentrations of (**A**) GSH and (**B**) GSSG, (**C**) the ratio of GSH to GSSG, (**D**) the concentrations of NADPH and (**E**) NADP^+^, and (**F**) the ratio of NADPH to NADP^+^. Results are represented as mean ± SE for *n* = 3. Means with different letters are significantly different at *p <* 0.05, according to Duncan’s multiple range test.

**Figure 5:**
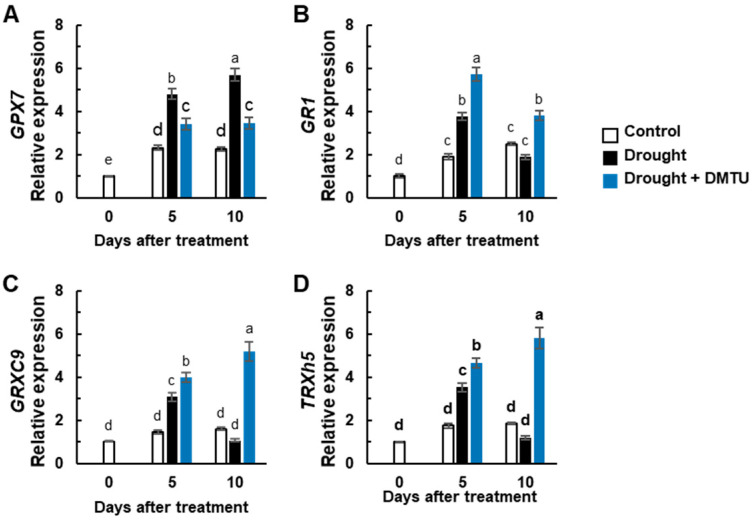
The changes of expression of redox regulating genes in the leaves of *Brassica napus* under control, drought, or drought with dimethylthiourea (Drought + DMTU) treatments for 10 days. The expression of (**A**) *glutathione peroxidase 7* (*GPX7*), (**B**) *glutathione reductase 1* (*GR1*), (**C**) *CC-type glutaredoxin 9* (*GRXC9*), and (**D**) *thioredoxin-h5* (*TRXh5*). The transcriptional levels in control plants at day 0 were set to 1. Results are represented as mean ± SE for *n* = 3. Means with different letters are significantly different at *p <* 0.05, according to Duncan’s multiple range test.

The concentration of reduced glutathione (GSH) was depressed, but oxidized glutathione (GSSG) was largely increased by the drought-alone treatment compared to the control. However, both concentrations were unchanged in the DMTU treatment under the drought condition (Figure 4A,B). The reduction of the GSH/GSSG ratio under drought was alleviated by DMTU treatment under the drought condition (Figure 4C). Significant increases in NADPH and NADP^+^ concentrations were observed in drought with or without DMTU treatment (Figure 4D,E). A significant decrease in the NADPH/NADP^+^ ratio at day 5 recovered to control levels in the presence of DMTU under the drought condition on day 10 but not in drought-alone condition (Figure 4F). The expression of the redox sensors, thioredoxin-h5 (*TRXh5*) and CC-type glutaredoxin 9 (*GRXC9*), and the glutathione reductase encoding gene (*GR1*), was significantly enhanced in drought with or without DMTU at day 5. Their expressions were suppressed by drought alone but continued to increase in the DMTU treatment under drought at day 10 (Figure 5B–D). The expression of the glutathione peroxidase encoding gene, *GPX7,* was enhanced overall in the drought-alone treatment (Figure 5A).

### 3.5. Heatmap Analysis and PCA of the Targeted ROS System, Redox Signaling, and Hormone Metabolism

**Figure 6:**
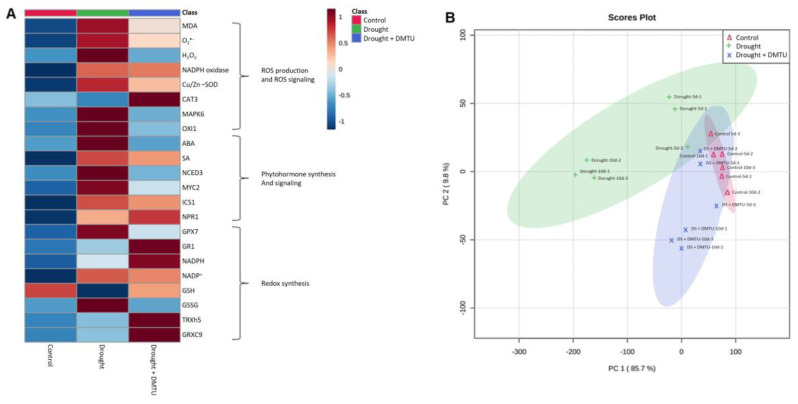
The heatmap analysis (**A**) and the principal component analysis (PCA) score plots (**B**) in the changes of the identified metabolites or gene expression levels in the reactive oxygen species (ROS) system-related, hormone, and redox signaling-related metabolism in the leaves of *Brassica napus* under control, drought, or drought with dimethylthiourea (Drought + DMTU) treatments for 10 days. (**A**) red indicates a positive effect, whereas blue indicates a negative effect. (**B**) pink, green, and purple colors indicate control, drought, and drought with DMTU treatments, respectively.

Metabolites or gene expressions significantly changed in the drought condition with or without DMTU compared to the corresponding control group. Heatmap analysis and PCA score plots analyzed in each category are shown in Figure 6. Heatmap analysis on the ROS system and hormone metabolism-related metabolites and gene expressions were highly increased by drought but alleviated by DMTU treatment (Figure 6A). Heatmap analysis on redox signaling showed an increase in the reduced redox forms, GSH and NADPH, and redox-related genes, *GR1*, *TRXh5*, and *GRXC9*, in the DMTU treatment under the drought condition compared to that of drought alone (Figure 6A). The results of the PCA score plot showed a clear separation among all treatments. The results of the PCA score plots accounted for 85.7% and 9.8% of the total variance of principal components (PC)1 and 2, respectively, and indicated that the drought alone and DMTU groups were segregated (Figure 6B).

### 3.6. Correlations among Treatment-Responses of Physiological Parameters

**Figure 7:**
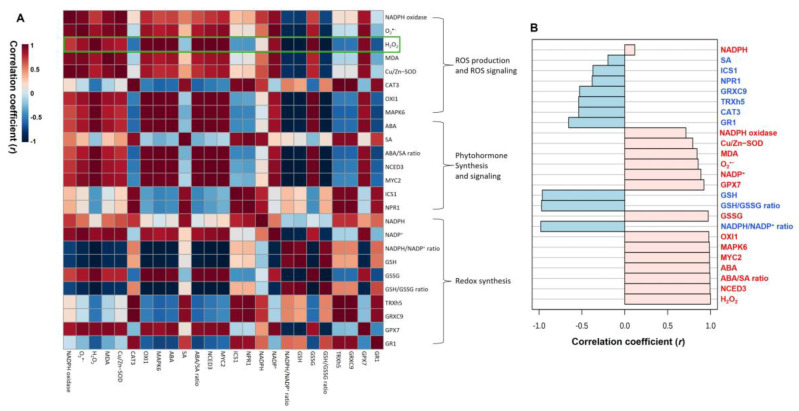
Heatmap responses of Pearson’s correlation coefficient (*r*) among the identified metabolites or gene expression levels (**A**) in the leaves of *Brassica napus* under control, drought, or drought with dimethylthiourea (Drought + DMTU) treatments at day 10. (**B**) The factors correlated with H_2_O_2_ concentration. Red and blue indicate positive and negative correlation coefficients. Color intensity is proportional to the correlation coefficients.

To further examine the functional implications and correlations of the identified metabolites or gene expression levels as affected by drought with or without DMTU treatment, we created Pearson correlation coefficients among ROS, ROS signaling, redox status, redox signaling, and phytohormones (Figure 7). A positive correlation was observed between ROS, ROS signaling, ABA synthesis, or ABA signaling-related genes, all negatively correlated with redox status. The expression of SA synthesis or signaling-related genes was positively correlated with redox signaling-related genes (Figure 7A). A comparative analysis of the factors related to H_2_O_2_ content (presented by the green box) suggested a positive correlation with the expression of ROS signaling-related genes (*MAPK6* and *OXI1*), redox-oxidized forms (GSSG and NADP^+^), ABA synthesis or signaling-related genes (*NCED3* and *MYC2*), or *GPX7*, and a negative correlation with GSH, GSH/GSSG ratio, NADPH/NADP^+^ ratio, or *GR1* (Figure 7B).

## 4. Discussion

### 4.1. DMTU-Mediated H_2_O_2_-Responsive Antioxidant System and ROS Signaling

Drought treatment by decreasing daily irrigation over 10 days reduced leaf water availability, and the recorded value of the leaf water potential (*Ψ_w_*) was −1.2 MPa, corresponding to a 2.5-fold decrease compared to that of the control leaves (Figure 1A). The *Ψ_w_* value was similar to that measured after 14 days of drought treatment in a different *B. napus* cultivar [2] and the range of −1.02 to −1.26 MPa measured over three days under polyethylene glycol (PEG)-induced drought stress [1]. The decrease in *Ψ_w_* was responsible for the induction of oxidative stress, as evidenced by the enhanced level of lipid peroxidation (Figure 1B) and an accumulation of ROS (O_2_^•−^ and H_2_O_2_) (Figure 2D,E). Lipid peroxidation and ROS accumulation, together with a decreasing *Ψ_w_*, have been commonly observed and thus considered symptomatic drought-induced stress events [1,2,17]. The level of ROS in plant cells is tightly regulated via coordination between ROS production and turnover [11,12,16] and determines the function of ROS as destructive or defensive signal transduction molecules [2,5,8,9]. In the present study, the drought-responsive increase in O_2_^•−^ and H_2_O_2_ was concomitant with the enhanced expression of NADPH oxidase, as reported previously in drought studies [2,17,35,36]. Drought results in a rapid decline in the internal CO_2_/O_2_ ratio of plants by stomatal closure in photosynthetic tissue, leading to hydrogen peroxide (H_2_O_2_) production in the peroxisomes through photorespiration [5]. ROS generation occurs as a result of the incomplete reduction of oxygen in the metabolic pathway and electron leakage [5,9], which is catalyzed by plasma membrane-localized nicotinamide adenine dinucleotide phosphate hydrogen (NADPHs) oxidases, cell wall peroxidases (POXs), and amine oxidase [11,37]. In the present study, the drought-enhanced expression of *NADPH oxidase* was concomitant with an increase in O_2_^•−^ and H_2_O_2_ concentrations (Figure 2 and Figure 7), which were positively correlated with each other (Figure 7). Plant NADPH oxidases (respiratory burst oxidase homologs, RBOHs) constitute a family of enzymes and are involved in several essential processes in enzymatic ROS-generating systems [38,39] and catalyze the production of the reactive oxygen ion superoxide [40]. However, the expression of *CAT3* responded negatively to the H_2_O_2_ concentration in antagonism with that of *NADPH oxidase* and *Cu/Zn-SOD* (Figure 2A–C and Figure 7). ROS (especially H_2_O_2_) are involved in signal transduction pathways to regulate the level of ROS production for maintaining homeostasis. One of the important signaling pathways acting on abiotic stress stimuli is the MAPK cascade. In the present study, drought treatment over 10 days prominently enhanced the expression of *MAPK6* (Figure 2F), accompanied by the enhanced expression of NADPH oxidase (Figure 2A). Several studies have shown that MAPKs act downstream of ROS and further positively regulate NADPH oxidase for ROS production [41,42]. Similarly, Xing et al. [43] reported that salt stress-induced *MAPK5* up-regulated the *Fe-SOD* gene, further generating ROS. In this study, the expression of *OXI1*, encoding an oxidative signal-induced serine/threonine kinase [15], was also highly activated by drought treatment (Figure 2G). H_2_O_2_-responsive enhancement of OXI1 protein kinase activity has been defined [15,44], and OXI1 is required for full activation of *MAPK3* and *MAPK6* [15]. Our previous studies have shown that H_2_O_2_ accumulation is concurrent with enhanced expression of OXI and MAPK6 [2,18]. It thus suggests that drought-induced ROS (especially H_2_O_2_) activate *MAPK6* and *OXI1* signaling, which are involved in ROS production and signaling under drought conditions.

Several ROS scavengers, such as imidazole, tiron, and dimethylthiourea (DMTU), have been known to mediate abiotic stress alleviation. Among these chemicals, DMTU has been studied in great detail in terms of its direct role in ROS trapping [45] and is an inducer of antioxidant enzymes and their encoding genes [31,46], and a regulator of secondary metabolism [33], which are involved in stress tolerance. The present data demonstrated that DMTU under drought significantly alleviated drought-induced lipid peroxidation (Figure 1B) and ROS accumulation (especially for H_2_O_2_) (Figure 2D,E) by suppressing *NADPH oxidase* and *Cu/Zn-SOD* expression and by activating *CAT3* (Figure 2A–C). This was accompanied by a depression of ROS signaling-related protein kinases (*OXI1* and *MAPK6*) (Figure 2F,G and Figure 6). Furthermore, correlation analysis indicated that the H_2_O_2_ concentration affected by drought with or without DMTU was tightly associated with *OXI1* and *MAPK6* (Figure 7B). These data thus suggest that DMTU may be effectively involved in ROS scavenging by regulating H_2_O_2_-dependent downstream ROS production and oxidative burst signaling.

### 4.2. DMTU-Mediated Changes in ROS-Hormonal Interaction

The interaction between ROS and hormones is known to play a pivotal role in regulating plant stress responses and has been reported [2,17,18,19]. For instance, the enhanced level of H_2_O_2_ caused by either drought treatment or exogenously supplied H_2_O_2_ activates the ABA biosynthesis gene (*NCED3*), leading to ABA accumulation and promoting H_2_O_2_ generation [2,18]. On the other hand, drought-induced ABA triggers the activation of H_2_O_2_ generation and increases cytosolic Ca^2+^ level closure [47,48] by the ABA-dependent activation of NADPH oxidase [19,24]. In the present study, the responses of endogenous H_2_O_2_ to drought with or without DMTU (Figure 2E) were very similar to those of the endogenous ABA level (Figure 3A and Figure 7), in accordance with the hypothesis that ROS (particularly H_2_O_2_) is part of ABA signaling [9,27,28]. Furthermore, the drought-responsive increase in the ABA level (Figure 3A) coincided with the enhancement of *MAPK6* and *OXI1* expression (Figure 2F,G), which was consistent with the up-regulation of ABA synthesis (*NCED3*) and ABA signaling (*MYC2*) (Figure 3D,E). In addition, our recent studies have shown that ABA accumulation, which occurred during the late drought period when H_2_O_2_ accumulation was prominent [2] and caused by drought or exogenously applied H_2_O_2_ [18], was responsible for the enhancement of *MAPK6* and *OXI1* expression. In the present study, drought-induced ABA-mediated *MAPK6* and *OXI1* were remarkably suppressed by DMTU treatment (Figure 2G,F and Figure 6A). Similarly, exogenous ABA-induced calcium-dependent transcription of *MAPK7* was depressed by ROS scavenger, such as imidazole, triton, and DMTU [32]. Together these results clearly indicate that the endogenous level of H_2_O_2_ and ABA may form a positive feedback loop in regulating oxidative burst signaling. In addition to ABA, a drought-responsive increase in SA level was also obvious, especially on day 5 under drought alone, but also on day 10 under drought with DMTU (Figure 3B) in accordance with SA synthesis (*ICS1*) and SA signaling (*NPR1*) (Figure 3F,G and Figure 6). The DMTU-mediated enhancement of SA level and SA-related gene expression coincided with the depression of ABA-induced *MAPK6* and *OXI1* (Figure 2 and Figure 3), indicating an antagonistic interaction between ABA and SA in an H_2_O_2_-dependent manner. The endogenous H_2_O_2_ level altered by drought with or without DMTU was positively related to the ABA level and the expression of ABA-related genes, while it was negatively related to SA responses (Figure 7). In our previous studies, antagonistic shifting from an ABA- to an SA-mediated process contributed to drought tolerance by regulating proline metabolism [17] and sucrose accumulation [49]. The studies based on the time course of drought intensity have shown that an increase in the SA level and the expression of SA-related genes enhanced at the early phase is part of the acclamatory process in response to mild stress intensity, whereas H_2_O_2_-activated ABA accumulation with an antagonistic depression of SA responses leads to severe drought symptoms at the late phase of drought [2]. These results suggest that DMTU-induced, SA-mediated antagonistic depression of ABA-dependent ROS production and oxidative burst signaling (*MAPK6* and *OXI1*) could be important in alleviating drought stress symptom development.

### 4.3. DMTU-Mediated Hormonal Regulation of NADPH- and GSH-Based Redox

In plant cells, oxidative damage occurs when the ROS generated by plant stress stimuli cannot be scavenged enough. Among ROS detoxifying systems, the primary pathway is the ascorbate-glutathione (AsA-GSH) pathway, which is known as the Asada–Halliwell pathway. This pathway requires energy in the form of NAD(P)H, which determines redox status in cells depending on the stress-induced modification of the metabolism [14,18,50]. The activity of ascorbate peroxidase, which converts H_2_O_2_ into the water with the help of AsA as an electron donor, was highly activated at 5 days, then largely down-regulated (data not shown), in accordance with previous results in drought-stressed white clover [51]. The present study focused on the GSH-based pathway in which drought-induced alteration was more prominent. Stress-enhanced H_2_O_2_ is reduced to H_2_O by ascorbate, while reduced GSH is converted to glutathione disulfide (GSSG) upon oxidation with two other GSH molecules [52]. The resultant oxidized glutathione (GSSG) is recycled back to GSH by glutathione reductase (GR) using NADPH as the reductant [53,54], thereby maintaining the redox potential of GSH. In the present study, drought significantly decreased the GSH pool (Figure 4A), which might be attributed to an insufficient sulfate supply caused by decreased mineral uptake [54] and the limiting role of the GSH synthesis enzyme (glutathione synthetase) [55]. Furthermore, drought caused a shift from the reduced GSH form toward a more oxidized disulfide form (i.e., GSSG) (Figure 4A–C), which was accompanied by the enhanced expression of the glutathione peroxidase (GPX)-encoding gene, *GPX7* (Figure 5A), along with an enhanced H_2_O_2_ concentration (Figure 2E). This agreed with the suggestion that GPX was strongly activated under a higher stress intensity, leading to the cell oxidizing process of GSH to GSSG conversion [56]. Underlining the H_2_O_2_-dependent ABA-activated ROS production and signaling as described-above, drought-induced GSH oxidation via GPX activation can be regulated in an H_2_O_2_-dependent ABA-mediated manner. Similarly, GPX is expressed predominantly when intracellular H_2_O_2_ is over-produced [57] through the activation of ABA insensitive 2 (ABI2), which is upstream of the ABA signaling pathway, under drought stress [58,59]. In addition, it has been reported that the GPX1 and GPX6 promoters contain ABA-related elements [60], and ABA treatment enhances the activity of GPXs [61]. On the other hand, in the treatment of drought in the presence of DMTU, the drought-enhanced GSSG was largely decreased by depressing *GPX7* with an antagonistic activation of the glutathione reductase (GR) encoding gene, *GR1* (Figure 4B and Figure 5A,B), thereby maintaining the GSH/GSSG ratio (Figure 4C). NADPH is used as a cofactor by GR to reduce GSSG to GSH and putatively by thioredoxin reductase to reduce oxidized thioredoxin [62]. Indeed, in the treatment of drought with DMTU, the reduction of GSSG toward GSH coincided with the enhancement of the NADPH level (Figure 4D) and *GR1* expression (Figure 5B), thereby recovering the NADPH/NADP^+^ ratio (Figure 4F). This maintenance of reducing potential in GSH- and NADPH-based redox (Figure 4 and Figure 6) also corresponded with the DMTU-enhanced SA level, especially at day 10 when the antagonistic depression of the ABA level and signaling strongly occurred (Figure 3 and Figure 7A). A proper GSH level has a key influence in controlling SA signaling molecule accumulation [63]. Moreover, the DMTU-enhanced SA level and signaling led to the recovery of glutaredoxin 9 (*GRXC9*) and thioredoxin-h5 (*TRXh5*) expression, which was markedly depressed by drought imposition (Figure 5C,D). Several studies have shown that SA is involved in modulating GSH- and/or NADPH-based redox [17,64,65] by up-regulating TRXh5 and GRXC9, which are essential for SA-mediated signaling transcriptional responses [30,65]. Therefore, this suggests that DMTU under the drought condition plays a significant role in NADPH- and GSH-based redox homeostasis by activating SA-mediated GR1 but suppressing *GPX7* expression with enhanced *TRXh5* and *GRXC9* expressions in the antagonism between ABA and SA.

## 5. Conclusions

The hormonal interaction with ROS (especially H_2_O_2_) is a primary regulatory system in drought stress responses and tolerance mechanisms. The present data are the first to report that DMTU (as a ROS scavenger) alleviates drought-induced oxidative stress in an antagonistic interaction between ABA and SA in rapeseed leaves. The physiological significance of DMTU in alleviating the drought-induced cell oxidation process is characterized by the following: (1) an activation of H_2_O_2_ scavenging leading to a depression of ROS signaling-related protein kinase (*OXI1* and *MAPK6*), (2) DMTU-induced, SA-mediated antagonistic depression of ABA-dependent ROS production and the expression of *MAPK6* and *OXI1*, and (3) SA-mediated *GR1* activation and *TRXh5* and *GRXC9* expression leading to NADPH- and GSH-based redox homeostasis. However, future studies are necessary to define the threshold where the endogenous H_2_O_2_ level shifts from an ABA- to an SA-mediated regulatory pathway (or vice versa), which might be an important determinant for the function of ROS as a positive secondary signal or an inducer of cell damage.

## Data Availability

Data is contained within the article and its Supplementary Materials.

## Supplementary Materials

The following supporting information can be downloaded at: https://www.mdpi.com/article/10.3390/antiox11112283/s1, Table S1: Specific primers used for RT-qPCR.
