# Supplementary material for: Dimethylthiourea Alleviates Drought Stress by Suppressing Hydrogen Peroxide-Dependent Abscisic Acid-Mediated Oxidative Responses in an Antagonistic Interaction with Salicylic Acid in Brassica napus Leaves
Source: Antioxidants (Basel). 2022 Nov 18;11(11):2283. doi: 10.3390/antiox11112283 (PMC9687642; doi:10.3390/antiox11112283)
Supplement: Supplementary file 1 [file antioxidants-11-02283-s001.zip › antioxidants-2007037-supplementary.pdf]

Supplementary Table S1. Specific primers used for RT-qPCR.

Target gene	GenBank Number	Forward sequence (5´-3´)	Reverse sequence (5´-3´)
BnNADPH oxidase	XM013788801	TGAGACGAGGGAATTCAGGAAC	GCTTCTCGTGGGAAGATCCG
BnCu/Zn-SOD	AY970822	TGCTAATCGTCATGCTGGAG	CTCCCTTTCCAAGGTCATCA
BnCAT3	NM001316190.1	GATCCTGCGGATGAGGATAA	AAGCAGCTTGTCATCCGAGT
BnOXI1	XM013843315	GCCACCAACTACCACAGGAT	CCCAAGCAATGACAAAACCT
BnMAPK6	XM_013884849.2	GCTAGCTCCATGGGACAGAG	GAGCAGTTGGTGGTGGATTT
BnNCED3	XM_013880797	GGAGTGCTTCTGCTTCCATC	TTCGAGGTTGACTTGCTCCT
BnMYC2	XM013880351	ACCAAACGTCTCGAAAATGG	TGTCAACGAGCAAGAGGATG
BnICS1	XM013887885	TCAATCCCAGAACGAGATCC	GACAGAAACCTTCGGATGGA
BnNPR1	EF613226.1	TGAGAACATTGCCAAGCAAG	CAACAGCAAAATGGAGAGCA
BnGRXC9	XM013796108	GAGAACGCGGTTATGGTGAT	TGGCGCCAATCTTCTCTAGT
BnTRXh5	XM013811982	GAAGTCTGGAGCGAGAAGAT	CGATCTTGAAGAAGACAACG
BnGPX7	XM009139799	GGATATCAAGCGGTTCGTGT	GTCGTGGGAGGGTATCTCTG
BnGR1	AF255651	CTGATTGGTGGGTTTTGCTT	ACCTTGGCACCATTGTTAGC
BnActin7	XM013867490	GATTCCGTTGCCCTGAAGTA	GCGACCACCTTGATCTTCAT
